# Study of the dissolved organic matter (DOM) of the Auzon cut-off meander (Allier River, France) by spectral and photoreactivity approaches

**DOI:** 10.1007/s11356-020-09005-7

**Published:** 2020-05-03

**Authors:** Davide Palma, Mohamad Sleiman, Olivier Voldoire, Aude Beauger, Edith Parlanti, Claire Richard

**Affiliations:** 1 Université Clermo Auvergne, CNRS, SIGMA-Clermont ICCF, 63000 Clermont-Ferrand, France; 2 Université Clermont Auvergne, CNRS, GEOLAB, 63000 Clermont-Ferrand, France; 3 Université Bordeaux, CNRS, UMR EPOC, 33405 Talence, France

**Keywords:** Riverine DOM, Absorption, Fluorescence, Reactive species, Photoreactivity, Correlations

## Abstract

Wetlands are recognized for the importance of their hydrological function and biodiversity, and there is now a consensus to protect and restore them as well as to complete the knowledge on their functioning. Here, we studied the dissolved organic matter (DOM) of a wetland composed of the Auzon cut-off meander, the Allier River, the alluvial fluvial flow, and watershed aquifer. Water was sampled at different locations, in spring, summer, and autumn. For each sample, DOM was characterized for its chemical and optical properties and its photooxidant capacity through its ability to generate DOM triplet excited states (^3^DOM*) and singlet oxygen upon simulated solar light exposure. UV-visible and fluorescence indices revealed that DOM was mainly microbial-derived whatever the sampling sites with spatial and temporal variations in terms of aromaticity (5.5–22%), specific UV absorbance at 254 nm (0.28–2.82 L m^−1^mgC^−1^), ratio of the absorbance at 254 and 365 nm (4.6–10.8), fluorescence index (1.35–166), and biological index (0.812–2.25). All the samples generated ^3^DOM* and singlet oxygen, rates of formation of which showed parallel variations. Using principal component analysis (PCA), we found positive correlations between the sensitizing properties of DOM samples and parameters associated to the abundance of low molecular weight and low absorbing chromophores. Moreover, the parameter variation across the wetland reinforced the hydrological movements observed in a previous study, suggesting that these parameters could be used as water connection tracers.

**Electronic supplementary material:**

The online version of this article (10.1007/s11356-020-09005-7) contains supplementary material, which is available to authorized users.

## Introduction

In the past, wetlands were considered as unhealthy areas and largely drained leading to a loss of about 64 to 71% of natural wetlands since 1900 because of human activities (Davidson 2014). Moreover, during the last decades, pollution intensification led to water body’s contamination with effects on aquatic ecosystems, human health, productive activities, water system reliability, and operating costs for water use (Gleick 1998; Sutton et al. 2013). Today, the importance of their hydrological function and biodiversity has been recognized, and there is a consensus to protect and restore them. Actions have therefore been implemented in order to preserve or restore wetlands, to complete the knowledge on their functioning, and to communicate about the necessity of maintaining that kind of environments.

Dissolved organic matter (DOM) that consists of water-soluble organic chemicals deriving from the decomposition of plants and living organisms is an important constituent of wetlands. DOM has different roles in freshwater systems. It has an effect on the speciation, transport, and availability of chemical elements and pollutants. Besides, DOM absorbs solar light and an important and largely documented property of DOM components is their capacity to degrade organic pollutants under solar light exposure (Shang et al. 2015). This property is based on their ability to generate reactive species under irradiation (Zepp et al. 1977; Cooper and Zika 1983; McCabe and Arnold 2016).

Our first objective in this work was to explore the relationships between the spectral properties of DOM and its capacity to generate reactive species under irradiation. Some correlations were reported in the literature. Parameters connected to the amount of DOM (organic carbon content, absorbance, or intensity of fluorescence at specific wavelengths) were found to be positively correlated to the formation rate of oxidant species such as singlet oxygen (^1^O_2_) and triplet excited states (^3^DOM*) (Coelho et al. 2011; Peterson et al. 2012; Timko et al. 2014; McCabe and Arnold 2016) while those linked to DOM quality (average molecular weight of DOM components, antioxidant activity) negatively correlated to the quantum yields of formation of these species (Dalrymple et al. 2010; McKay et al. 2017).

Spectral properties of DOM give useful information about the chemical characteristics and origins of their components. The specific UV absorbance at 254 nm (SUVA_254_) gives the aromatic carbon content of DOM (Weisshar et al. 2003). The spectral slope (*S*_275–295_) and the ratio of the absorbance at 254 and 365 nm (E_2_/E_3_) are both linked to the relative size of DOM molecules (De Haan and De Boer 1987; Peuravouri and Pihlaja 1997). Fluorescence indices such as the fluorescence index (FI), the biological index (BIX), and the humification index (HIX) are related to the terrestrial versus aquatic source of DOM (Stedmon and Markager 2005; Coble 2007; Huguet et al. 2009; Birdwell and Engel 2010), and the humification degree (Zsolnay et al. 1999). On the other hand, the rate of reactive species photoproduction can be obtained using probe molecules (Rosario-Ortiz and Canonica 2016).

**Fig. 1:**
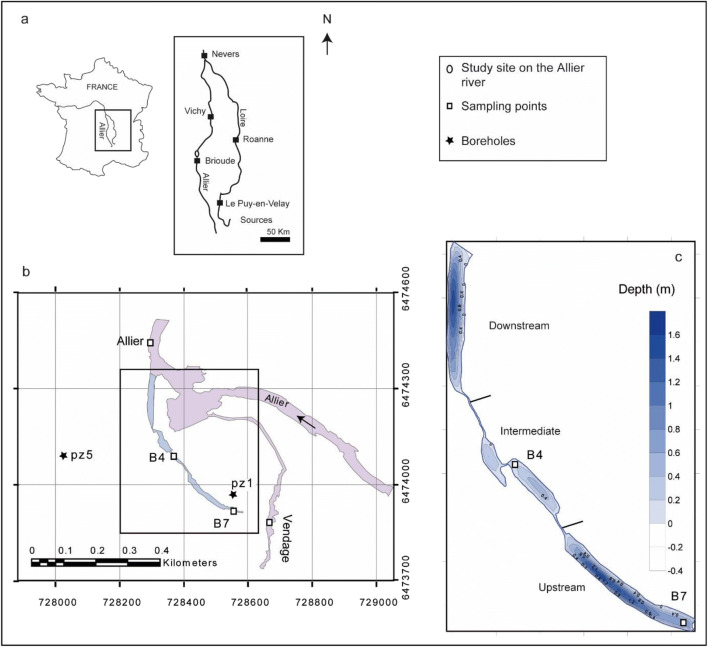
**a** Location of the Auzon site. **b** Scheme of the Auzon site. **c** The three zones of the Auzon cut-off meander. Sampling points are noted as open squares and boreholes as black stars

The present study was carried out on a wetland including the Auzon cut-off meander, the Allier River and its tributary Vendage, as well as two aquifers, the alluvial fluvial flow and the watershed ones (Fig. 1). This site looked very appropriate to the study because it offers diversity regarding some parameters among those affecting quality and quantity of DOM: hydrologic conditions, vegetation cover, organic matter inputs, and photochemical and biological degradation processes (Sobek et al. 2007; Gao et al. 2018; McCullough et al. 2019; Queimaliños et al. 2019). This wetland DOM is therefore expected to vary quantitatively and qualitatively with the location and could also change over the season (McCabe and Arnold 2016).

Previous results showed that the connection degree is high between the cut-off meander and both the main stream and alluvial aquifer (Beauger 2008). Indeed, a non-negligible supply of the cut-off meander by the alluvial groundwater is observed, especially in its downstream part. Our second objective, here, was to use the results on the variations of DOM in the site in terms of chemical and spectral characteristics to better understand the hydrological movements of the site.

To achieve this work, surface water was taken at 6 places: three in the Auzon cut-off meander, one in the Allier River and two in boreholes, in spring, summer, and fall. Chemical and spectral characteristics of DOM as well as their ability to generate ^3^DOM* and ^1^O_2_ were determined for all samples across seasons and locations. Then, principal component analysis (PCA) was performed to explore the relationships between all the variables.

## Materials and methods

### Site description and samplings

The study area is a cut-off meander of the Allier River, a tributary of the Loire River situated in the temperate zone (Massif central, France) (Fig. 1a). A complete description is given in SI text 1. The Auzon cut-off meander was formed when the Allier River captured a gravel-pit and modified the course of the stream (Beauger 2008; Beauger et al. 2015) (Fig. 1b). The cut-off meander approximates 560 m length and is divided into three distinct zones (Fig. 1c): (1) the upstream zone disconnected from the river and similar to a pond, with an important development of macrophytes; (2) the intermediate zone corresponding to a geomorphological riffle characterized by the alternation of lentic and lotic areas with dominant mineral substrates; (3) the downstream zone characterized by bedload deposits coming from the river and macrophytes. For the present study, we retained two sampling sites of surface water in the cut-off meander: B4 situated in the intermediate zone in the middle of the lentic area where solar irradiation is high, and B7 situated in the uppest part of the upstream zone where the riparian forest induced less light penetration. Moreover, two of the boreholes: one on the right bank (PZ1) that collected the alluvial fluvial flow at 5 m depth and the other on the left bank (PZ5) that collected the groundwater of the watershed at 12 m depth were also retained as well as one in the Allier River, and another in the Vendage River, a small tributary of the Allier River. Samples were collected in March, July, and October 2018 allowing us to study seasonal variations in the ability of DOM to produce reactive species. They were taken from surface water (between 10 and 30 cm) in pre-rinsed glass bottles and transported in ice to the laboratory. All samples of natural water underwent a 2 steps vacuum filtration at 1.2 μm and at 0.45 μm using cellulose membranes filters. The filtered samples were placed in cap sealed Pyrex-glass bottles and stored at 4 °C until uses.

### Chemicals

Furfuryl alcohol (FFA, 98%) and 2,4,6-trimethyphenol (TMP, certified reference material) were purchased from Sigma-Aldrich and used as received. Acetonitrile and methanol for HPLC were from Carlo-Erba and VWR, respectively. All the solutions were prepared in water purified using a reverse osmosis RIOS 5 and Synergy (Millipore) device (resistivity 18 MΩ cm, DOC < 0.1 mg L^−1^).

### Chemical analyses

Dissolved organic carbon (DOC) concentrations were measured using a Shimadzu TOC-L analyzer. Phthalic acid was used for calibration. Analyses were done in triplicate. The reproducibility of measurements was of 2% maximum. Major anions (Cl^−^, Br^−^, NO_3_^−^, NO_2_^−^) were analyzed using a Thermofisher Dionex DX120 HPLC.

### Spectral measurements

Absorption spectra were recorded using a Varian Cary 3 spectrophotometer in a 5-cm path length quartz cell. Spectra were scanned from 200 to 800 nm at 1-nm interval and corrected for the ultrapure water reference. A_280_ was obtained by dividing the absorbance at 280 nm by 5. SUVA_254_ was obtained by dividing the decadic absorption coefficient at 254 nm (m^−1^) by the DOC concentration (mg L^−1^). It gives the aromatic carbon content of DOM from the relationship proposed by Weisshar et al. (2003):

1 $$ \mathrm{Percent}\ \mathrm{aromaticity}=6.52\times {\mathrm{SUVA}}_{254}+3.63 $$

The ratio E_2_/E_3_ was obtained by dividing the absorbance at 254 by that at 365 nm (Peuravouri and Pihlaja 1997). It is an indicator of the aromaticity and the average molecular weight (Mw) of DOM. Mw was calculated using the relationship proposed by Lou and Xie (2006) in the case of humic and fulvic acids isolated from a forest soil and from Suwannee River (International Humic Substances Society):

2 $$ \mathrm{Mw}=0.315\times \exp \left(4.96/\left(-1.72+{\mathrm{E}}_2/{\mathrm{E}}_3\right)\right) $$

The spectral slope S_275_–_295_ was calculated as the slope of the linear regression of ln(a_λ_) on λ, between λ = 275 and 295 nm, where a_λ_ is the napierian absorption coefficient expressed in m^−1^. ....

Fluorescence spectra were scanned on a Perkin Elmer LS-55 spectrofluorometer in a 1-cm path length quartz cell. Both slit widths were set to 10 nm, and the scanning speed at 240 nm min^−1^. When necessary, the solutions were diluted to have an absorbance ≤ 0.10 at 250 nm. Emission spectra were recorded between 250 and 600 nm for different excitation wavelengths (260, 310, and 370 nm). FI that is an indicator of relative contribution of aquatic/microbial and terrestrial DOM sources was obtained by dividing the emission intensity at 450 nm by that at 500 nm with excitation at 370 nm (McKnight et al. 2001). BIX informs on the importance of freshly autochthonous DOM, it was obtained by dividing the ratio of emission intensity at 380 nm to that at 430 nm with excitation at 310 nm (Huguet et al. 2009).

### Irradiation experiments

TMP and FFA were used independently as chemical probes to estimate the formation rate of ^3^DOM* and ^1^O_2_ in the water samples, respectively (Rosario-Ortiz and Canonica 2016 and references therein). The processes are the following. ^3^DOM* produced after reactions 3 and 5 undergoes deactivation through reaction 5, transfers its energy to O_2_ through reaction 6, or oxidizes TMP by reaction 7. ^1^O_2_ is deactivated by the solvent in reaction 8 and oxidizes FFA through reaction 9. 6-Hydroxypyran-3-one (P) is the main oxidation product of FFA by singet oxygen (chemical yield of 85%) (Haag et al. 1984).

3 $$ \mathrm{DOM}+\mathrm{h}\upnu \to {\kern0.28em }^1{\mathrm{DOM}}^{\ast } $$

4 $$ {}^1{\mathrm{DOM}}^{\ast}\to {\kern0.28em }^3{\mathrm{DOM}}^{\ast } $$

5 $$ {}^3{\mathrm{DOM}}^{\ast}\to \mathrm{DOM} $$

6 $$ {}^3{\mathrm{DOM}}^{\ast }+{\mathrm{O}}_2\to \mathrm{DOM}+{}^1{\mathrm{O}}_2 $$

7 $$ {}^3{\mathrm{DOM}}^{\ast }+\mathrm{TMP}\to {\mathrm{DOM}}_{+\mathrm{H}}+{{\mathrm{TMP}}_{-\mathrm{H}}}^{.} $$

8 $$ {}^1{\mathrm{O}}_2+\mathrm{solvent}\to {\mathrm{O}}_2 $$

9 $$ {}^1{\mathrm{O}}_2+\mathrm{FFA}\to \mathrm{P} $$

According to these reactions, the rate of TMP consumption, R^TMP^, is equal to:

10 $$ {\mathrm{R}}^{\mathrm{T}\mathrm{MP}}={\mathrm{R}}_{\mathrm{a}}\times \kern0.5em {\Phi}_{\mathrm{T}}\times {\mathrm{k}}_7{\left[\mathrm{TMP}\right]}_0/\left({\mathrm{k}}_{\mathrm{d}}+{\mathrm{k}}_7{\left[\mathrm{TMP}\right]}_0\right) $$

where R_a_ is the rate of light absorption by DOM (Einstein L^−1^ s^−1^), Φ_Τ_, the quantum yield of ^3^DOM^*^ formation, k_5_, the rate constant of reaction between ^3^DOM^*^ and TMP (reaction 7), [TMP]_0_, the initial concentration of TMP and k_d_, the first order rate constant decay of ^3^DOM^*^ by deactivation (reaction 5) and reaction with oxygen (reaction 6), the sum k_5_ + k_6_.

The rate of R^P^ is equal to:

11 $$ {\mathrm{R}}^{\mathrm{P}}=0.85\times {\mathrm{R}}_{\mathrm{a}}\times {\Phi}_{\mathrm{SO}}\times {\mathrm{k}}_9{\left[\mathrm{FFA}\right]}_0/\left({\mathrm{k}}_8+{\mathrm{k}}_9{\left[\mathrm{FFA}\right]}_0\right) $$

where Φ_SO_ is the polychromatic quantum yield of singlet oxygen formation, k^’^, the rate constant of reaction between singlet oxygen and FFA (reaction 9, 1.2 × 10^8^ s^−1^) (Haag et al. 1984), [FFA]_0_, the initial concentration of FFA and k_d_^’^, the first-order rate constant of deactivation of singlet oxygen (reaction 8, 2.5 × 10^5^ s^−1^ in water) (Wilkinson et al. 1995).

R_a_ was obtained using the relationship:

12 $$ {R}_a=\sum \limits_{\lambda_1}^{\lambda_2}{I}_0^{\lambda }\ \left(1-{10}^{- A\lambda}\right)\times \Delta \lambda \times 1000{N}^{-1} $$

where I_0_^λ^ is the amount of photons at λ reaching the solution, averaged between λ−2.5 nm and λ+2.5 nm (photons cm^−3^ nm^−1^ s^−1^); A_λ_ is the averaged absorbance of the solution at λ calculated between λ−2.5 nm and λ+2.5 nm for a path length equal to 1.4 cm; λ_1_ and λ_2_ are the integration limits, N is the number of Avogadro, and Δλ is the wavelength interval chosen at 5 nm.

Solutions containing FFA (100 μM) or TMP (50 μM) were put in a cylindrical reactor (1.4 cm, i.d.) made out of Pyrex-glass open to air and irradiated in a device equipped with six polychromatic tubes (Sylvania, F15 W/350BL) emitting within the wavelength range 300–450 nm (maximum emission at 365 nm, Fig. SI-2). A radiometer QE65000 from Ocean optics was used to measure the spectral distribution of the light emitted by the tubes. In addition, the use of metamitron as a chemical actinometer (Kouras et al. 2011) allowed us to get the amount of light received by the solution per nm and second. Fifteen milliliters of solutions were irradiated for each experiment. Aliquots (500 μL) were removed at selected intervals and immediately analyzed by HPLC. Irradiations had a duration of 30 min where FFA and TMP reached a conversion extent comprised between 10 and 20%. TMP and P concentrations were monitored by HPLC using a Waters apparatus equipped with a 2695 separation module, a 2996 photodiode array detector and a reverse phase Nucleodur, Macherey-Nagel C_8_ column (5 μm, 150 mm × 4.6 mm). A flow rate of 1 mL min^−1^ was used for all analyses and the eluent was a mixture of 20% methanol and 80% water acidified with orthophosphoric acid (0.1%) for the experiments with FFA while a mobile phase of 50% acetonitrile and 50% acidified water was used for the experiments with TMP. All experiments and HPLC analyses were carried out in duplicate. The initial rates of TMP disappearance (R^TMP^) and of P formation (R^P^) were obtained by plotting the concentration of TMP and P over irradiation time, respectively.

### Statistical analyses

Statistical analyses and tests discussed in this work (Pearson correlation, principal component analysis (PCA), and hierarchical clustering) were performed with the R statistical software (R version 3.6.1, R Foundation for Statistical Computing). PCA is a powerful tool used for reducing the dimensionality of a set of variables and for identifying the main axes of variance within a dataset. PCA thus allows for easy data exploration and visualization by transforming it into fewer dimensions that nonetheless retains most of the information and brings out strong trends and patterns (Lever et al. 2017). PCA can also help identify clusters in the data which are grouped using hierarchical clustering. Distinct PCAs were conducted for DOM optical indices with and without sensitizing parameters. Pearson correlation coefficients were reported for all correlations where data were normally distributed. *P* values < 0.01 were considered statistically significant.

## Results and discussion

### Chemical and spectral properties of DOM

Tables 1 and SI-1 summarize results obtained from chemical and optical analyses of the samples and Fig. SI-3 presents the absorption spectra. The pH varied within a very narrow range around the neutrality. Nitrite, nitrate, and chloride were found as major ions. Nitrite concentration was less than 0.165 mg L^−1^ and nitrate concentration less than 2.7 mg L^−1^ except in the Allier River where it reached 5.3 mg L^−1^ in March and in the Vendage River with values up to 21 mg L^−1^. These levels are too low to have a significant effect on the photochemical experiments in our irradiation conditions (Vione et al. 2005). DOC ranged between 3.28 and 36.0 mg C L^−1^ with lowest values measured for B4 and B7 in March and October and PZ1 in October and highest values for the Vendage River and PZ5 in July. DOC of Allier River samples varied moderately over the year ranging from 4.74 to 6.39 mg C L^−1^, while, for the other sites, a strong DOC increase was measured in July with values exceeding those measured in March and October by factors comprised between 2.9 and 5.3. In July 2018, the water surface of the cut-off meander and Vendage River was covered with aquatic plants. The presence of these aquatic plants may explain the high DOC increase in July considering that macrophytes can release organic matter through photosynthetic processes (Reitsema et al. 2018). It was reported that released DOC mostly consists of small (< 1000 Da) molecules that include amino acids and simple sugars (Søndergaard 1981).

**Table 1:** Chemical and optical characteristics of water samples (see experimental part). *A_280_ was measured in a 5-cm path length cell

Site	Season	pH	DOC (mg C L^−1^)	A_280_^*^	SUVA (L m^−1^ mg C^−1^)	Aromaticity (%)	S_275–295_ (nm^−1^)	E_2_/E_3_	Mw (kDa)	BIX	FI
Allier	March	7.47	5.5	0.516	2.60	21	0.0126	5.22	1.3	0.812	1.35
	July	7.29	6.4	0.629	2.82	22	0.0138	5.24	1.3	0.988	1.46
	Oct.	6.98	4.7	0.394	2.36	19	0.0147	5.38	1.2	0.936	1.38
B4	March	7.30	4.9	0.128	0.78	8.7	0.0156	6.01	1.0	1.10	1.54
	July	7.13	14	0.150	0.30	5.6	0.0148	5.57	1.1	2.25	1.59
	Oct.	7.40	4.9	0.209	1.15	11	0.0127	5.12	1.4	1.06	1.6
B7	March	6.93	3.3	0.139	1.10	11	0.0152	5.43	1.2	1.09	1.58
	July	6.85	15	0.569	0.49	6.8	0.0134	4.6	1.8	1.02	1.55
	Oct.	6.91	4.6	0.224	1.26	12	0.0117	5.52	1.2	1.09	1.6
Vendage	March	7.82	9.6	0.594	1.78	15	0.0139	6.38	0.91	0.956	1.45
	July	7.87	36.0	0.578	0.44	6.50	0.0139	5.75	1.1	0.989	1.49
	Oct.	7.71	10.5	0.581	1.55	13.7	0.0117	4.56	1.8	1.11	1.54
PZ1	March	7.04	6.66	0.234	1.08	10.7	0.0166	9.3	0.61	1.02	1.54
	July	7.11	14.5	0.271	0.55	7.22	0.0146	6.50	0.89	1.25	1.60
	Oct.	7.26	3.36	0.143	1.25	11.8	0.0152	7.06	0.80	1	1.57
PZ5	March	7.15	9.97	0.094	0.30	5.59	0.0177	10.8	0.54	1.22	1.71
	July	7.10	33.8	0.309	0.280	5.46	0.0156	8.12	0.68	1	1.62
	Oct.	7.41	6.4	0.112	0.53	7.09	0.0176	8.19	0.68	1.16	1.66

The UV-visible absorption spectra were typical of DOM showing a featureless decreasing exponential decay from 200 to 500 nm and a more or less pronounced shoulder between 250 and 300 nm attributable to aromatic moieties (Fig. SI-3). Except in July, the DOM of the two riverine sites, Allier and Vendage, exhibited the highest A_280_ and SUVA_254_ values (0.394–0.629 and 2.82–1.55 mg C L^−1^ m^−1^) and the highest aromatic content (22–15.2%). These values were however in the low range of those found for other river samples (Weisshar et al. 2003). The lowest aromatic content was measured for PZ5 water samples (5.06–7.09%), and it was comparable to those reported for some oceanic samples (Weisshar et al. 2003). DOM of PZ1 samples exhibited higher values (7.22–11.8%) closer to those of the two cut-off meander samples. SUVA_254_ dropped significantly in July for B4, B7, PZ1, and Vendage River samples, indicating that the organic matter released in summer was less aromatic than in the other seasons. In contrast, for PZ5, SUVA_254_ did not change although a significant DOC increase showing that in this case the nature of DOM was more constant over the year.

Variations of S_275–295_ among samples paralleled those of E_2_/E_3_ as often reported (Helms et al. 2008). Highest values were measured for PZ5 and PZ1 samples for which we could therefore estimate that calculated Mw were low (0.543–0.889 kDa) compared to those of the other water samples (1.0–1.75 kDa). The low Mw values in PZ1 and PZ5 can be explained by the adsorption of larger macromolecules to the soil constituents during the percolation/transfer processes.

Emission spectra recorded for excitation at 260, 310, and 370 nm were used to calculate indices. All the samples exhibited an intense emission at 340 nm and a humic-like peak (λexc 260 nm/λem 380–460 nm) (Coble 2007) (Fig. SI-4). Values of fluorescence indices 1.35 < FI < 1.7 and 0.81 ≤ BIX ≤ 2.2 showed that all the samples predominantly contained DOM of autochthonous origin with freshly produced organic matter (McKnight et al. 2001; Huguet et al. 2009; Birdwell and Engel 2010). With lowest FI values, the Allier and Vendage River samples had the highest terrestrial organic matter content in accordance with the highest aromaticity and the highest A_280_ values.

To sum up, although DOM was mainly microbially derived whatever the sampling sites, chemical and spectral properties varied among the samples that can be ranked. DOM of riverine sampling sites Allier and Vendage are on the top of the scale for their absorbance, aromaticity, average molecular weight and highest terrestrial organic matter content while DOM from PZ5 is clearly on the bottom of the scale. DOM from B4 and B7 lay in the medium of the scale and PZ1 approaches B4 and B7 for SUVA_254_, and aromaticity but PZ5 for average molecular weight.

### Sensitizing properties

The use of TMP and FFA as chemical probes had for objective to compare the ability of the different samples to generate ^3^DOM* and ^1^O_2_ under irradiation and thus to get information on their ability to photodegrade micropollutants.

**Fig. 2:**
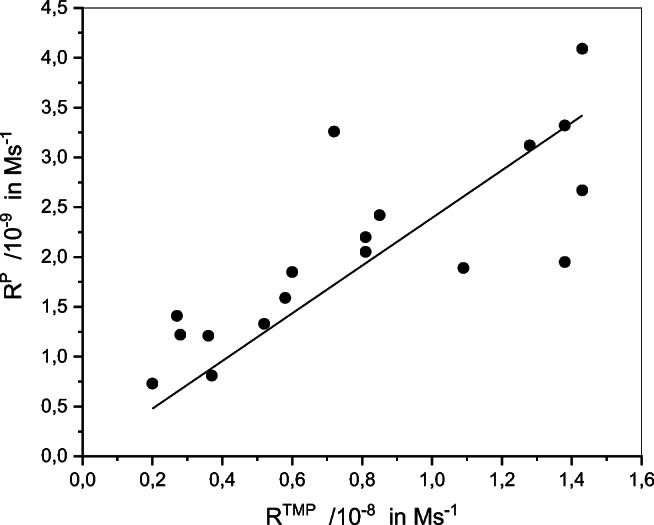
Plot of the rate of P formation (R^P^) vs the rate of TMP consumption (R^TMP^)

All the water samples were able to photodegrade TMP and to oxidize FFA into P (Fig. SI-5) in accordance with the photochemical generation of ^3^DOM* and ^1^O_2_, respectively (reactions 3-9). Kinetic data are reported in Table 2. R^TMP^ ranged from 0.2 × 10^−8^ to 1.43 × 10^−8^ M s^−1^ for TMP (5 × 10^−5^ M) and thus varied by a factor of 7 among samples while the rate of P formation (R^P^) ranged from 0.73 × 10^−9^ and 4.09 × 10^−9^ M s^−1^ for FFA (10^−4^ M) varying by a factor 5.6. According to Eq. 10, R^TMP^ is proportional to the rate of light absorption by the water samples, the quantum yield of ^3^DOM* formation and the percentage of ^3^DOM* trapped by TMP at 5 × 10^−5^ M. If we make the hypothesis that this percentage is quite constant among water samples, then we get, R^TMP^ = Cte × R_a_ × Φ_Τ_. On the other hand, according to Eq. 11, R^P^ is equal to Cte’ × R_a_ × Φ_SO_. The linear increase of R^TMP^ with R^P^ (*R* = 0.80, *p* < 0.01, Fig. 2) as already observed (McKay et al. 2017) confirmed that Φ_Τ_ variations paralleled those of Φ_SO_ in accordance with the direct involvement of ^3^DOM* in the formation of ^1^O_2_.

**Table 2:** Results of the kinetic data processing (see experimental part)

Site	Season	R_a_/10^−6^ (Einstein L^−1^ s^−1^)	R^TMP^/10^−8^ (M s^−1^)	R^TMP^/R_a_	R^P^/10^−9^ (M s^−1^)	Φ_SO_
Allier	March	3.48	0.810	0.0023	2.05	0.010
	July	4.30	1.38	0.0032	3.32	0.013
	October	2.67	1.38	0.0052	1.95	0.012
B4	March	0.77	0.370	0.0048	0.810	0.015
	July	1.03	0.520	0.0050	1.33	0.021
	October	1.48	0.280	0.0019	1.22	0.014
B7	March	0.94	0.200	0.0021	0.730	0.011
	July	2.14	0.850	0.0040	2.42	0.019
	October	1.36	0.270	0.0020	1.41	0.017
Vendage	March	3.43	1.09	0.0032	1.89	0.0078
	July	3.51	1.43	0.0041	4.09	0.019
	October	4.36	0.720	0.0016	3.26	0.012
PZ1	March	1.03	0.810	0.0079	2.20	0.030
	July	1.63	1.28	0.0077	3.12	0.031
	October	0.75	0.600	0.0080	1.85	0.040
PZ5	March	0.34	0.360	0.011	1.21	0.050
	July	1.51	1.43	0.0095	2.67	0.029
	October	0.41	0.580	0.014	1.59	0.063

R^TMP^ and R^P^ were the highest for Allier and Vendage Rivers in July and October, and for PZ1 and PZ5 in July and the lowest for B4 and B7 samples in March. As PZ1 and PZ5 did not contain the more highly absorbing DOM, it means that variations of R^TMP^ and R^P^ did not parallel those of light absorption. Therefore, in a second step, we corrected R^TMP^ and R^P^ for the rate of light absorbance and got access to the R^TMP^/R_a_ ratios that are proportional to Φ_T_ and to Φ_SO_ according to our mechanistic hypotheses. This gives a better view of the capacity of DOM chromophores to generate reactive species and allows us to rank them in terms of sensitizing capacity. Moreover, our data can be compared with the ones reported in the literature for other DOM sampled in various aquatic systems. Ranging from 0.010 to 0.063, Φ_SO_ fall within the values previously published 0.010–0.039 for Sandvik et al. (2000) and 0.009–0.055 for Peterson et al. (2012).

### Correlations among variables

**Fig. 3:**
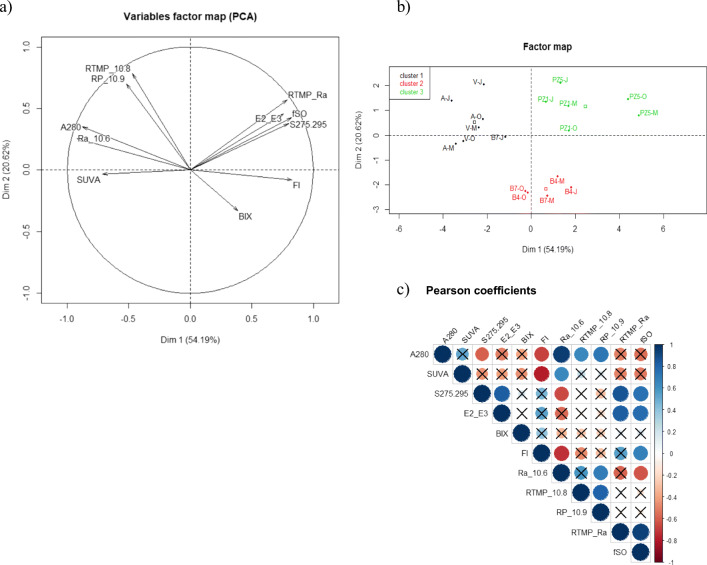
Variable factor map (**a**) and individual factor map (**b**) and Pearson coefficients (**c**) from PCA obtained from 11 variables: A_280_, SUVA, E_2_/E_3_, S_275_/_295_, BIX, FI, Ra, R^TMP^, R^P^, R^TMP^/Ra, and Φ_SO_. The symbols not barred with a black cross correspond to *p* values < 0.01

PCA was run with 11 variables: A_280_, SUVA, E_2_/E_3_, S_275_/_295_, BIX, FI, Ra, R^TMP^, R^P^, R^TMP^/Ra, and Φ_SO_. This analysis revealed that the first two principal factors (PC1 and PC2) explained 54.19% and 20.62% of the total variance, respectively and thus 74.81% of the total variance (Fig. 3). The score plot of the different samples and the Pearson correlation coefficients are also shown in Fig. 3. Variables E_2_/E_3_, S_275_/_295_, R^TMP^/Ra, Φ_SO_, and FI occurred in the positive part of PC1 axis and were positively correlated. The correlation coefficient of Φ_SO_ with R^TMP^/Ra, S_275_/_295_, E_2_/E_3_ and FI was equal to 0.93, 0.74, 0.77 and 0.67 respectively with *p* < 0.01. It was often reported in the literature that f_TMP_ (= Φ_T_ × k_5_/k_d_) that is close to our R^TMP^/Ra ratio as well as Φ_SO_ were positively correlated with E_2_/E_3_ and thus increased when Mw of DOM decreased (Dalrymple et al. 2010; McKay et al. 2017; Maizel and Remucal 2017). Mw of DOM increases with the abundance in high molecular weight molecules. The big macromolecules are expected to reduce the sensitizing properties (i) through internal deactivation processes or (ii) by scavenging of reactive species (Boyle et al. 2009). A_280_, Ra, and SUVA are three variables connected to DOM absorbance and are logically correlated (*R* = 0.63–0.98, *p* < 0.01). The position of these variables in the negative part of PC1 axis indicated that high DOM absorbance negatively affected the sensitizing properties. This appears reasonable as high DOM absorbance is associated to a high aromatic content and/or great abundance of highly conjugated molecules. It can be also seen that FI was negatively correlated with SUVA, A_280_ and Ra (*R* = − 0.79, − 0.72, − 0.72 with *p* < 0.01) in accordance with the lower absorptivity/molecular weight of fresh autochthonous DOM compared to allochthonous DOM. PC1 seemed thus associated to the relative abundance of low/high molecular weight and low/high absorbing chromophores.

PC2 was positively correlated to R^TMP^ and R^P^ and negatively to BIX. Riverine DOM samples except Vendage October, and piezometer DOM samples had moderate positive loadings while cut-off meander DOM samples moderate to low negative loadings. PC2 seemed therefore associated to the conjugated character of chromophores. In riverine samples, the conjugated molecules have an allochthonous origin while in piezometer samples they come from soil organic matter and microorganisms. In the cut-off meander, the organic matter is fresh and poorly conjugated in average. The cut-off meander DOM of microbial/macrophytes origin had little better sensitizing properties than Riverine DOM of terrestrial origin either due to the lower concentration of highly absorbing compounds or to better inherent sensitizing properties (Felcyn et al. 2012; Bodhipaksha et al. 2015; Maizel and Remucal 2017; McCabe and Arnold 2017).

### Links between DOM properties and hydrological movements

**Fig. 4:**
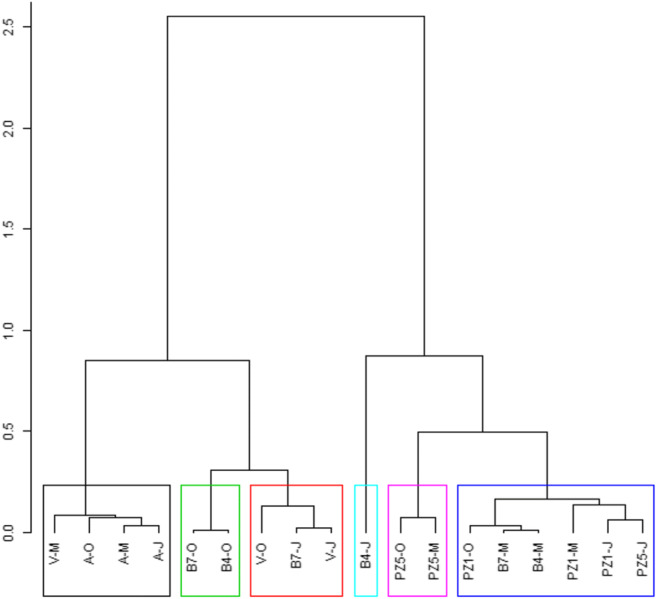
Hierarchical clustering obtained from PCA obtained from 6 variables: A_280_, SUVA, E_2_/E_3_, S_275_/_295_, BIX, and FI

Related to this study, it appeared that B4, B7, and PZ1 showed similarities in terms of chemical, spectral and sensitizing properties of DOM. To better visualize this, we run a PCA based on the 6 spectral variables: A_280_, SUVA, E_2_/E_3_, S_275_/_295_, BIX, and FI. The hierarchical clustering is shown in Fig. 4. A first cluster was composed by DOM of Riverine Allier March, July and October and Vendage March, a second by DOM of B4 and B7 in October, a third by DOM of Vendage in July and October and B7 in July, a fourth by PZ5 March and October and a fifth by PZ1 March, July, and October, B4 and B7 in March, and PZ5 in July. B4 July showed a very singular behavior and was alone. These observations were reinforced by a previous research that had underlined the supplying of cut-off meander by the Allier River. This supply takes place by the surface connection at the downstream confluence and by an underground connection through the upstream paleochannel (Quenet et al. 2019). In this latter case, B7 is the closest location to the paleochannel arrival. B7 is fed by the Allier River with 64% of contribution (Quenet et al. 2019). This supplies lead to more similar physical and chemical conditions between B4, B7 and PZ1 in particular during winter and spring (Quenet et al. 2019).

The sampling site PZ5 that is situated on the left bank is much less affected by the Allier River. Indeed, the alluvial groundwater was identified to supply the cut-off meander during low flow at the left bank. Thus, PZ5, influenced by the alluvial aquifer, is apart considering both the hydrodynamic functioning (Quenet et al. 2019) and the results of this study (DOM and DOC results). Moreover, PZ sampling points present some differences compared to the surface sampling points as groundwater has a percolation origin. At last, DOM river Allier all over the year and Vendage in March were apart as they are running water crossing forests and fields. They are enriched in organic matter from soil compared to the other DOM and are more aromatic. DOM Vendage in July and October were close to DOM of B4 October and B7 October suggesting connections.

To conclude, this Auzon site presents a diversity of DOMs that were characterized by their chemical and spectral properties and their ability to generate reactive species across the year. Three sub-groups were identified: DOM of running rivers, DOM of standing water cut-off meander and DOM of aquifers. As expected, parameters variations were linked to hydrologic movements, vegetation cover and organic matter inputs. Analysis of the data also informs on the hydrological functioning of this wetland.

## Electronic supplementary material

ESM 1 (DOCX 569 kb)
