# Supplementary material for: Study of the dissolved organic matter (DOM) of the Auzon cut-off meander (Allier River, France) by spectral and photoreactivity approaches
Source: Environ Sci Pollut Res Int. 2020 May 3;27(21):26385–94. doi: 10.1007/s11356-020-09005-7 (PMC7332482; doi:10.1007/s11356-020-09005-7)
Supplement: Supplementary file 1 — (DOCX 569 kb) [file 11356_2020_9005_MOESM1_ESM.docx]

Supporting Information

Study of the dissolved organic matter (DOM) of the Auzon cut-off meander (Allier river, France) by spectral and photoreactivity approaches

Davide Palma^a^, Mohamad Sleiman^a^, Olivier Voldoire^b^, Aude Beauger^b^, Edith Parlanti^c^, Claire Richard^a*^

^a^Université Clermont Auvergne, CNRS, SIGMA-Clermont, ICCF, F-63000 Clermont-Ferrand, France

^b^Université Clermont Auvergne, CNRS, GEOLAB, F-63000 Clermont-Ferrand, France

^c^Université Bordeaux, CNRS, UMR EPOC, Talence F-33405, France

* Corresponding author: Claire Richard

Claire.richard@uca.fr

Tel: +33 (0)4 73 40 71 42

Fax: +33 (0)4 73 40 77 00

**Texte 1 : Full description of the site**

The study area is a cut-off meander of the Allier River, a tributary of the Loire River situated in the Massif central (France) (**Fig. SI-1a & b**) in the southern part of a graben (in the small subsidence basin of Brioude). The area is about 400 m a.s.l and 160 km from the source. The study area presents a temperate continental climate with relatively hot summers (air temperatures up to 24.6°C and 23.9°C in mean respectively in July and August) and cold winters (mean temperatures around -2 and -3°C from December to February; “Météo-France” n.d.). Annual precipitations are moderate (692 mm on average) due to a shelter effect which affects the valley and most of the events occur between May and October (423 mm in cumulative precipitation; “Météo-France” n.d.). Mountainous areas of the upper Allier catchment are more humid with a total precipitation around 2000 mm mainly occurring during winter and late spring and minimums during summer (around 570 mm). The basin is composed of metamorphic and basaltic bedrock and filled with Oligocene sand and clay deposits. The agricultural activities (grazing and cereals production) remain the main anthropogenic pressures. Most of the water resource of the area is located in the modern alluvial formations that constitute the aquifer of the Allier River alluvial groundwater. This resource is intensely exploited for the supply of drinking water but not on the study site. This unconfined aquifer extends over a width of between 0.5 and 2 km and a thickness of 15 m in the most favorable areas, as for the Auzon cut-off meander.

In 1989. the Auzon cut-off meander was formed when the Allier River captured a gravel-pit and modified the course of the stream. The upper part of the channel was rapidly in-filled by sand and gravels; since then, only the downstream end of the cut-off meander remains connected to the main stream conferring it the first stage in oxbow cycle. The cut-off meander analyzed over its approximate 560 m length was divided into three distinct zones (**Fig. SI-1c**): 1) the upstream zone disconnected from the river and similar to a pond. with an important development of macrophytes (197 m length. 13 m width. maximum depth around 1.80 m. slope = 0.01%; 2) the intermediate zone corresponding to a geomorphological riffle characterized by the alternation of lentic and lotic areas with dominant mineral substrates (176 m length, width ranging from 30 cm to 8 m, maximum depth around 0.40 m, slope = 0.39%); 3) the downstream zone characterized by bedload deposits coming from the river and macrophytes (188 m length. 17 m width. maximum depth around 1.60 m. slope = 0.06%). Since the first studies in 2007, the site’s monitoring is extending to water quality analyses by sampling surface water within the cut-off meander and the Allier River to complete ecological investigations in 2012. In 2014, SOAHAL Observatory (Système d'Observation d'une Annexe Hydraulique de l'Allier) was set with the implantation of deep observation boreholes to monitor alluvial groundwater and complete the water quality monitoring.

**Fig. SI-2** Fluorescent tubes irradiance

**Fig. SI-3** : UV-visible spectra of the different samples

**Fig. SI-4** : Emission spectra of samples upon excitation at 260 nm. Black line for March. red line for July and blue line for October.

**Fig SI-5** : A) Typical consumption profile of TMP when irradiated in the presence of cut-off meander DOM and B) formation of photoproduct of FFA

A)

B)

**Table SI-1** : Concentration of major ions (mg.L^-1^) in the different samples

| March | Cl^-^ | Br^-^ | NO_2_^-^ | NO_3_^-^ |
| --- | --- | --- | --- | --- |
| Allier | 10.9 | 0.0 | 0.014 | 5.3 |
| B4 | 31.5 | 0.0 | 0.014 | 0.62 |
| B7 | 15.9 | 0.0 | 0.011 | 2.7 |
| Vendage | 56.5 | 0.0 | 0.165 | 21 |
| PZ1 | 45.1 | 0.0 | 0.010 | 1.3 |
| PZ5 | 78.7 | 0.0 | 0.011 | 1.8 |

| July | Cl^-^ | Br^-^ | NO_2_^-^ | NO_3_^-^ |
| --- | --- | --- | --- | --- |
| Allier | 9.2 | 0.0 | 0.01 | 2.5 |
| B4 | 19.8 | 0.0 | 0.01 | 1.2 |
| B7 | 11.6 | 0.0 | 0.01 | 3.3 |
| Vendage | 52.4 | 0.0 | 1.19 | 16.0 |
| PZ1 | 10.8 | 0.0 | 0.01 | 2.2 |
| PZ5 | 35.0 | 0.0 | 0.02 | 3.9 |

| October | Cl^-^ | Br^-^ | NO_2_^-^ | NO_3_^-^ |
| --- | --- | --- | --- | --- |
| Allier | 10.1 | 0.0 | 0.01 | 1.5 |
| B4 | 19.1 | 0.0 | 0.01 | 0.2 |
| B7 | 15.2 | 0.0 | 0.01 | 1.9 |
| Vendage | 84.5 | 0.0 | 1.21 | 13.8 |
| PZ1 | 12.5 | 0.0 | 0.01 | 0.1 |
| PZ5 | 82.3 | 0.0 | 0.02 | 0.4 |
